# Translation, cross-cultural adaptation and psychometric evaluation of yoruba version of the short-form 36 health survey

**DOI:** 10.1186/s12955-015-0337-y

**Published:** 2015-09-14

**Authors:** Chidozie Emmanuel Mbada, Gafar Atanda Adeogun, Michael Opeoluwa Ogunlana, Rufus Adesoji Adedoyin, Adesanmi Akinsulore, Taofeek Oluwole Awotidebe, Opeyemi Ayodiipo Idowu, Olumide Ayoola Olaoye

**Affiliations:** 1 Department of Medical Rehabilitation, College of Health Sciences, Obafemi Awolowo University, Ile – Ife, Nigeria; 2 African Population and Health Research Center, Nairobi, Kenya; 3 Department of Physiotherapy, College of Medicine, University of Ibadan, Nigeria, Nigeria; 4 Department of Mental Health, College of Health Sciences, Obafemi Awolowo University, Ile – Ife, Nigeria; 5 Department of Physiotherapy, School of Basic Medical Sciences, College of Medical Sciences, University of Benin, Benin City, Nigeria

**Keywords:** Health-related quality of life, Yoruba SF-36, Translation, Cultural adaptation, Psychometric properties

## Abstract

**Background and objective:**

The Short-Form Health Survey (SF-36) is a valid quality of life tool often employed to determine the impact of medical intervention and the outcome of health care services. However, the SF-36 is culturally sensitive which necessitates its adaptation and translation into different languages. This study was conducted to cross-culturally adapt the SF-36 into Yoruba language and determine its reliability and validity.

**Methods:**

Based on the International Quality of Life Assessment project guidelines, a sequence of translation, test of item-scale correlation, and validation was implemented for the translation of the Yoruba version of the SF-36. Following pilot testing, the English and the Yoruba versions of the SF-36 were administered to a random sample of 1087 apparently healthy individuals to test validity and 249 respondents completed the Yoruba SF-36 again after two weeks to test reliability. Data was analyzed using Pearson’s product moment correlation analysis, independent *t*-test, one-way analysis of variance, multi trait scaling analysis and Intra-Class Correlation (ICC) at *p* < 0.05.

**Results:**

The concurrent validity scores for scales and domains ranges between 0.749 and 0.902 with the highest and lowest scores in the General Health (0.902) and Bodily Pain (0.749) scale. Scale-level descriptive result showed that all scale and domain scores had negative skewness ranging from −2.08 to −0.98. The mean scores for each scales ranges between 83.2 and 88.8. The domain scores for Physical Health Component and Mental Health Component were 85.6 ± 13.7 and 85.9 ± 15.4 respectively. The convergent validity was satisfactory, ranging from 0.421 to 0.907. Discriminant validity was also satisfactory except for item ‘1’. The ICC for the test-retest reliability of the Yoruba SF-36 ranges between 0.636 and 0.843 for scales; and 0.783 and 0.851 for domains.

**Conclusion:**

The data quality, concurrent and discriminant validity, reliability and internal consistency of the Yoruba version of the SF-36 are adequate and it is recommended for measuring health-related quality of life among Yoruba population.

## Introduction

Health-Related Quality of Life (HRQoL) is distinguished from quality of life in that it is concerned primarily with those factors that fall under the purview of health care providers and health care system [1]. HRQoL as a multi-dimensional concept describes the effect of diseases and illnesses on persons′ physical, social and mental well-being [2, 3] and it is important in estimating the efficacy of medical intervention on quality of life [4–6] and also to monitor community health [2, 3].

Literature is replete on various HRQoL measures or test batteries which can either be generic or disease-specific [2–5, 7–11]. Amid the various measurement tools, the Medical Outcomes Study (MOS) Short Form 36-item Health Survey (SF-36), is one of the most widely used generic measures of HRQoL with good psychometric properties and substantial data on its applicability in clinical and research settings [6, 9–14]. The SF-36 was derived from the original 245 items RAND MOS Questionnaire as a set of generic, coherent, and easily administered quality of life measures [9, 10, 15]. The 36 items health survey tool assesses eight health dimensions referred to as subscales, namely Physical Functioning (PF: 10 items), Role Limitations due to Physical Problems (RP: 4 items), Bodily Pain (BP: 2 items), General Health (GH: 5 items), Vitality (VT: 4 items), Social Functioning (SF: 2 items), Role Limitation due to Emotional Problems (RE: 3 items) and Mental Health (MH: 5 items) [9, 10]. These subscales’ scores are summarized into physical and mental composite domains [9, 10]. Individual SF-36 items are recoded, summed and transformed. The health concepts described by the SF-36 range in score from 0–100, with higher scores indicating higher levels of function or better health. Scores on the eight subscales can be used to compute a summary index of Physical Health Component (PC) and Mental Health Component (MC) respectively [9, 10].

The SF-36 has been employed to compare quality of life between different disease groups and populations [7, 16]. However, Cheung [17] opined that the SF-36 scores cannot be used to make valid inferences about racial/ethnic group differences when measurement equivalence is not provided, such differences might be due to item response bias rather than true differences in self-reported health. Non-equivalence can occur when differences in values, attitudes, language and overall world view cause respondents to respond to survey questions differently, leading to differential item functioning [18]. Therefore, the cultural sensitivity and bias of the SF-36 necessitates its adaptation and translation into different languages. Consequently, the SF-6 has been translated for use in both general and condition-specific populations in many languages such as Arabic [14], Chinese [19], Malay [20] and Persian [21] among others.

The SF-36 has been used by some Nigerian researchers [22–26], with two studies reporting content and criterion validity of its Yoruba translations among patients with hypertension [25] and low-back pain [26] respectively. However, cross-cultural adapted versions of the SF-36 in indigenous Nigerian languages based on internationally accepted guidelines seems not to be available for referencing. Therefore, this study was conducted to cross-culturally adapt the SF-36 into Yoruba language and determine its reliability and validity. Yoruba is one of the major Nigerian languages spoken in Southwest Nigeria, and also in countries like Benin and Togo. In addition, there is a pocket of Yoruba population especially in the UK, Brazil and the USA [27, 28].

## Methods

The study protocol was approved by the Ethical Review Committee, Institute of Public Health, College of Health Sciences, Obafemi Awolowo University, Ile-Ife, Nigeria (IPHOAU/12/156). A total of 1087 (657 males and 430 females) individuals comprising of students, workers and other residents of Ile-Ife, Osun state, Nigeria volunteered for this study, yielding a response rate of 98.8 % (i.e. 1087/1100). Informed consent was obtained from all the respondents. Eligible respondents were 18 years and older, literate in English and Yoruba languages and with no reported history of cognitive or mental impairment or current medical condition. A multistage sampling technique was employed in the study. Respondents from Obafemi Awolowo University, Ile-Ife comprised of students and staff. The students were recruited from four randomly selected students’ halls of residence (two for male and female students respectively). Every odd numbered room in each block in the halls of residence was sampled. Staff respondents were volunteers sampled from ten randomly selected departments using a fishbowl technique. Respondents who were resident outside the university community were recruited based on the World Health Organization guideline for conducting community surveys [29]. These respondents were randomly selected from five out of eleven political wards of Ile-Ife Local Government Area. Every odd numbered house was selected for survey.

Based on the International Quality of Life Assessment (IQOLA) Project, the English version of the SF-36 was translated into Yoruba language. The IQOLA project was established in 1991 with the goal of developing validated translations of a health status questionnaire as required for their use internationally in order to avoid bias in interpretation and adaptation [10, 19, 30].

The protocol was carried out in sequential order as highlighted by Fukuhara et al. [12]:

- Forward translation of the items and response choices of the English version of the SF-36 into Yoruba language by two native Yoruba speakers with fluency in English (Translator A and B). These translators are linguists and educators in Yoruba language at the University who worked independently to produce two initial Yoruba versions of the SF-36. Both translators were instructed, as described in the IQOLA protocol, to aim for conceptual rather than literal translation and to keep the language colloquial and compatible with a reading level of age 14 as described by Fukuhara et al. [12]. The translators were also asked to give difference translations for each response choice where possible.
- Harmonization and reconciliation of the two different translations was carried out. Another bilingual translator reviewed the items in the two Yoruba translated questionnaires in order to produce a single, reconciled and harmonized translation. Based on consensus among the translators, items were included in the reconciled translation. For items that were linguistically or culturally problematic, the translators decided on the most acceptable option after exhausting all reasonable options.
- Two natives who were literate in oral and written Yoruba language assessed the Yoruba consensus translation for comprehensibility and ambiguity on the difficulty and quality rating in terms of clarity, common language usage, and conceptual equivalence. A scale of 0 to 100 (0 means “not at all difficult” and 100 means “extremely difficult”) was used for difficulty rating, while quality rating was also rated on a scale of 0 to 100 (where 100 indicate perfection). First assessor rated the harmonized translation 70 and 80 % on difficulty and quality scale respectively, while the other assessor rating was 80 and 90 % for difficulty and quality scale respectively.
- The harmonized forward translation was back translated into English by two bilingual (English and Yoruba) professional translator for conceptual equivalence with the original source version.
- In order to validate the back translated English version by comparing it with the source English version, independent rating of the equivalence of the backward translations to the English version was carried out.
- Problematic items and response options were reconciled through an iterative procedure.
- The pre-final version of the Yoruba SF-36 was pilot tested among 32 individuals. The pilot test was aimed to explore the clarity and applicability of the translated Yoruba SF-36 in terms of perception, understanding of various terminologies used and interpretations. The results of the cognitive debriefing from the pilot study was used to further refine the pre-final version in terms of words used and the format or layout of the questionnaire.

The clustering and ordering of items in the translated Yoruba SF-36 was the same as the English version of the SF-36. However, in order to give the translated Yoruba SF-36 a conceptual equivalence to the English SF-36, arrangement and wording of some parts of the questionnaire were altered. The following cultural adaptations were made to the translated Yoruba SF-36:

i. Alphabetic numbering of the translated Yoruba SF-36 is not consistent with the English version. The alteration in the alphabetic numbering was because ‘C’ does not exist in the Yoruba alphabets, meanwhile alphabets such ‘Ẹ’ and ‘GB’ which are in the Yoruba does not exist in the English alphabets. For example, the first eight Yoruba alphabets are –a, b, d, e, ẹ, f, g, gb. Therefore, ‘d’ is the 3rd and ‘gb’ is the 8th Yoruba alphabet, hence the alphabetic numbering alteration of the translated Yoruba SF-36.
ii. Item 1 was rearranged in order to enhance its meaning in Yoruba language (This is because instruction comes before question).
iii. In question ‘3b’, “pushing a vacuum cleaner and bowling” were changed into “floor mopping and archery” respectively. Pushing a vacuum cleaner and bowling are uncommon activities in the study context. Therefore “floor mopping” was considered as an alternative moderate activity for “pushing vacuum cleaner”. On the other hand, bowling which refers to a number of sports or activities involving throwing a bowling ball towards a target, is strange to the Yoruba culture. However, archery which also refers to a sport involving shooting arrows at a target using a bow can easily be related with by the Yoruba people.
iv. ‘Blocks’ as a measure of distance in questions ‘3 h’ and ‘3i’ of the English SF-36 (which corresponds to ‘3gb’ and ‘3i’ in the Yoruba SF-36) were changed in the Yoruba SF-36 to ‘electric pole distance’. This is because blocks are less known as a descriptive measure of distance in the study setting compared with electric pole distance. However, the distance between two high tensions electric poles are commonly 50 m, which is not an equivalent of ′one block” that actually means a distance of 100 m. Nonetheless, it also known that distance between blocks are not fixed and varies widely in different setting.

Respondents completed both English and Yoruba versions of SF-36 as well as questions on socio-demographic variables. Thereafter, the respondents rated the English and Yoruba versions of SF-36 separately based on standard method of rating SF-36 Questionnaire recommended by IQOLA on the same day. Two weeks later, the respondents were asked to rate their quality of life on the Yoruba version of SF-36 again and the scores were compared with the initial rating.

Computation of the SF-36 involves, firstly, recoding of the pre-coded numeric values based on SF-36 scoring key for the required 35 out of the 36 items. Each item is recoded on a 0 to 100 range. Items “1, 2, 6, 8, 11b, 11d” with pre-coded numeric values 1 through 5, are recoded inversely to values of 100, 75, 50, 25, and 0 respectively. Items “7, 9a, 9d, 9e, 9 h” with pre-coded 1 through 6, are recoded inversely to values of 100, 80, 60, 40, 20 and 0 respectively. On the other hand, items “3a, 3b, 3c, 3d, 3e, 3f, 3 g, 3 h, 3i” with pre-coded numeric values, 1 through 3 are recoded in the same direction to values of 0, 50 and 100 respectively. Items in “4a, 4b, 4c, 4d, 5a, 5b, 5c” with pre-coded numeric values, 1 and 2 are recoded in the same direction to values of 0 and 100 respectively. Items in “10, 11a, 11c” with pre-coded numeric values, 1 through 5 are recoded in the same direction to values of 0, 25, 50, 75 and 100 respectively, while items in “9b, 9c, 9f, 9 g, 9i” with pre-coded numeric values, 1 through 6 are recoded in the same direction to values of 0, 20, 40, 60, 80 and 100 respectively.

Secondly, items in the same hypothesized scale are computed and averaged together to create the eight scale scores. The scales and the constituent items are 1) GH –“1, 11a, 11b, 11c, 11d”; 2) PF - “3a, 3b, 3c, 3d, 3e, 3f, 3 g, 3 h, 3i, 3j”; 3) RP – “4a, 4b, 4c, 4d”; 4) RE – “5a, 5b, 5c”; 5) SF – “6, 10”; 6) MH – “9b, 9c, 9d, 9f, 9 h”; 7) BP – “7, 8”; and 8) VT – “9a, 9e, 9 g, 9i”. Thirdly, scales in the same domain are computed and averaged together to create the two domain scores. The domains and the constituent scales are 1) PC – “GH, PF, RP, BP”; and 2) MC – “MH, RE, SF, VT”.

In order to determine the psychometric properties of the Yoruba version of the SF-36, it was hypothesized that items, scales and domain scores would correlate significantly (r >0.40) with the English SF-36. Based on correlation co-efficient (r) cut-off points for high (= > 0.70), moderate (0.4 - <0.7) and low (<0.40), high correlations (>0.70) were considered desirable because this would indicate good validity of the translated Yoruba SF-36 (Appendix).

### Data analysis

Descriptive statistics of scales and domains of the Yoruba version of SF-36 was determined by analyzing mean score, confidence interval, skewness and Kurtosis. Concurrent validity of the Yoruba SF-36 was determined by correlating scores of English and Yoruba versions of the SF-36 using Pearson’s product moment correlation. Intra class correlation (ICC) was used to determine the reliability (test-retest) of the Yoruba SF-36. Multi trait scaling analysis (i.e. item-scale correlations) was used to confirm item discriminant validity (i.e. correlations between each item and its hypothesized scale). Known-groups validity of Yoruba version of SF-36 was tested by comparing scale and domain scores by gender and age groups using independent *t*-test and One-way ANOVA respectively. Data was analyzed using SPSS (Statistical Package for Social Sciences) version 16.0. Alpha level was set at *p* < 0.05.

## Results

The respondents’ ages ranges between 18 and 70 years with the mean of 27.9 ± SD 9.48 years. The socio-demographic characteristics of the respondents are presented in Table 1. The respondents were mostly of the Yoruba tribe (95.8 %), single (67.8 %), Christians (41.3 %) and had tertiary education (83.8 %). The mean, confidence interval, skewness and Kurtosis of mean scores for the eight scales/dimensions of the Yoruba version of SF-36 are presented in Table 2. The result shows that the mean scores for the scales range between 83.2 and 88.8. The highest and lowest scores were observed in the MH (88.8) and RE (83.2). PC and MC domain scores was 85.6 ± 13.7 and 85.9 ± 15.4 respectively. The scale and domain scores yielded negative skewness ranging from −2.08− −0.98 on the Yoruba version of SF-36.

**Table 1:** Socio-demographic characteristic of the respondents (*n* = 1087)

Variables	Frequency	%
Age group		
18-24	475	43.80
25-34	447	41.12
35-44	79	7.27
45-54	51	4.69
≥ 55	35	3.22
Gender		
Male	657	60.4
Female	430	39.6
Marital status		
Single	737	67.8
Married	335	30.8
Widowed	10	0.9
Divorced	5	0.5
Ethnic group		
Hausa	9	0.8
Igbo	14	1.3
Yoruba	1041	95.8
Others	23	2.1
Religion		
Christianity	626	57.6
Islam	449	41.3
Traditional	12	1.1
Education level		
No formal	2	0.2
Primary	8	0.7
Secondary	166	15.3
Tertiary	911	83.8

**Table 2:** The mean score, standard deviation, confidence interval, Skewness and kurtosis of each scales and components (domains) of Yoruba SF-36 (*n* = 1087)

	Scale							Domain		
	GH	PF	RP	RE	SF	MH	BP	VT	PC	MC
Mean	86.9	86.1	85.1	83.2	84.6	88.8	84.8	86.8	85.6	85.9
SD	16.9	18.2	24.2	29.0	18.1	13.4	17.7	15.4	13.7	15.4
Confidence interval										
Lower	84.7	84.8	83.7	81.5	83.5	88.0	83.7	85.8	83.9	85.0
Upper	89.1	87.4	86.5	85.0	85.7	89.6	85.9 87.7	87.4	86.8	
Skewness	−2.08	−1.64	−1.56	−1.71	−1.06	−1.17	−1.27	−1.25	−0.98	−1.15
Kurtosis	4.76	2.17	1.68	1.90	0.42	1.23	1.24	0.98	0.19	0.87

Key: Scales: physical functioning (*PF*), role limitations due to physical problems (*RP*), bodily pain (*BP*), general health (*GH*), vitality (*VT*), social functioning (*SF*), role limitations due to emotional problems (*RE*), and mental health (MH). Domains: physical health component (*PC*) and mental health components (*MC*)

Table 3 shows the Pearson correlation analysis between respondents’ scores on the English and Yoruba SF-36 (concurrent validity). The correlation co-efficient (r) of the scales and domains ranges between 0.749 and 0.902. GH and BP had the highest (*r* = 0.902) and lowest (*r* = 0.749) correlation co-efficient respectively. Correlations between each item and its hypothesized scale (i.e. scale score computed from all other items in that scale as a test of item internal consistency) were all above 0.50, except for item 1 and GH (i.e. “In general, would you say your health is′) where *r* = 0.421. The highest item-scale correlation coefficient was between item 8 and other items on the BP sub-scale yielding a correlation co-efficient of 0.907 (i.e. “During the past four weeks, how much did pain interfere with your normal work -including both work outside the home and housework”). The details for item-scale correlations (discriminant validity) for Yoruba SF-36 is presented in Table 4. The result shows that items in VT, SF and MH scales had correlation scores greater than 0.23 with scales other than their hypothesized scales except the correlations between item 9a (“Did you feel full of pep?”) and each of RP (0.192) and PF (0.146). Correlations of item 11c (“I expect my health to get worse”) of GH with other scales were less than 0.3. Correlations of items in hypothesized GH scale with items in MH, BP and EF scales were less than 0.3 except correlations between 4b (“Accomplished less than you would like”) and GH; and 4c (“Were limited in the kind of work or other activities”) with each of MH and EF (Table 4).

**Table 3:** Pearson correlation analysis between respondents’ scores on the English and Yoruba SF-36 (concurrent validity) (*n* = 1087)

	r	*p*-value
Scale		
GH	0.902	0.001*
PF	0.845	0.001*
RP	0.813	0.001*
RE	0.838	0.001*
SF	0.845	0.001*
MH	0.811	0.001*
BP	0.750	0.001*
VT	0.801	0.001*
Domain		
PC	0.839	0.001*
MC	0.887	0.001*

Alpha level was set at *p* < 0.05; * indicate significance at *p* = 0.001; *r* = Pearson’s correlation coefficient

Key: Scales; physical functioning (*PF*), role limitations due to physical problems (*RP*), bodily pain (*BP*), general health (*GH*), vitality (VT), social functioning (*SF*), role limitations due to emotional problems (*RE*), and mental health (*MH*). Domains; physical health component (*PC*) and mental health components (*MC*)

**Table 4:** Item-scale correlations (discriminant validity) of the Yoruba SF-36 (*n* = 1087)

		GH	PF	RP	RE	SF	MH	BP	VT
GH	1	0.421	0.372	0.324	0.431	0.460	0.418	0.428	0. 437
GH	11a	0.749	0.411	0.283	0.329	0.364	0.331	0.347	0.327
GH	11b	0.789	0.339	0.283	0.284	0.420	0.365	0.280	0.376
GH	11c	0.565	0.265	0.128	0.013	0.155	0.143	0.216	0.139
GH	11d	0.780	0.297	0.225	0.214	0.303	0.314	0.294	0.491
PF	3a	0.237	0.635	0.411	0.358	0.456	0.326	0.317	0.334
PF	3b	0.242	0.625	0.373	0.255	0.391	0.388	0.342	0.342
PF	3c	0.264	0.665	0.395	0.255	0.376	0.359	0.338	0.343
PF	3d	0.158	0.742	0.317	0.320	0.240	0.303	0.264	0.279
PF	3e	0.195	0.577	0.272	0.275	0.353	0.325	0.305	0.324
PF	3f	0.122	0.590	0.207	0.206	0.320	0.218	0.285	0.230
PF	3 g	0.477	0.728	0.465	0.307	0.383	0.327	0.353	0.274
PF	3 h	0.250	0.748	0.405	0.366	0.319	0.266	0.283	0.280
PF	3i	0.139	0.559	0.291	0.299	0.353	0.264	0.266	0.248
PF	3j	0.159	0.571	0.283	0.284	0.293	0.322	0.332	0.285
RP	4a	0.219	0.347	0.536	0.269	0.158	0.169	0.141	0.205
RP	4b	0.323	0.440	0.754	0.401	0.384	0.293	0.282	0.275
RP	4c	0.193	0.430	0.659	0.390	0.369	0.372	0.282	0.321
RP	4d	0.056	0.287	0.641	0.409	0.309	0.195	0.208	0.217
RE	5a	0.252	0.287	0.337	0.692	0.344	0.340	0.274	0.342
RE	5b	0.246	0.262	0.387	0.720	0.387	0.315	0.296	0.341
RE	5c	0.291	0.274	0.385	0.780	0.391	0.399	0.258	0.379
SF	6	0.395	0.399	0.393	0.552	0.854	0.544	0.538	0.561
SF	10	0.360	0.383	0.421	0.427	0.870	0.586	0.541	0.563
MH	9b	0.433	0.400	0.400	0.428	0.478	0.785	0.423	0.645
MH	9c	0.418	0.512	0.395	0.409	0.516	0.741	0.552	0.576
MH	9d	0.487	0.255	0.228	0.260	0.488	0.738	0.499	0.623
MH	9f	0.396	0.239	0.277	0.388	0.513	0.770	0.453	0.641
MH	9 h	0.479	0.271	0.276	0.299	0.474	0.667	0.440	0.601
BP	7	0.437	0.433	0.357	0.468	0.603	0.608	0.805	0.620
BP	8	0.308	0.390	0.284	0.276	0.512	0.478	0.907	0.454
VT	9a	0.373	0.146	0.192	0.325	0.481	0.575	0.431	0.745
VT	9e	0.511	0.306	0.351	0.360	0.522	0.615	0.469	0.621
VT	9 g	0.403	0.419	0.373	0.447	0.500	0.681	0.542	0.731
VT	9i	0.391	0.421	0.304	0.361	0.542	0.697	0.493	0.776

For the known-groups validity of the Yoruba version of the SF-36 by gender and age, Table 5 shows the result of the independent *t*-test comparison of scales and domains by gender. The result showed that men had significant higher mean scores in GH (*p* = 0.022), RP (*p* = 0.054), RE (*p* = 0.013) and SF (*p* = 0.013) scales respectively. There were no significant gender differences in domain scores (*p* > 0.05). On the other hand, Table 6 shows the result of the One-way ANOVA comparison of scales and domains by age group. There were significant differences in the mean scores of the Yoruba SF-36 scales and domains (*p* < 0.05). The younger age group (18-24years) had significantly higher mean scale and domain mean scores (*p* < 0.05). A decline in mean scores with higher age was observed across the different scales and domains except within the age bracket 35–44 years where high mean scores were found (except for GH scale).

**Table 5:** Independent *t*-test comparison of scales and domains score of the Yoruba version of the SF-36 by gender

	Gender			
	Men	Women		
	x̄ ± SD (*n* = 657)	x̄ ± SD (*n* = 430)	t-cal	p-value
Scale				
GH	87.2 ± 14.7	86.4 ± 20.6	0.355	0.022*
PF	87.0 ± 17.8	84.7 ± 18.7	1.686	0.083
RP	86.1 ± 23.7	83.6 ± 25.0	1.659	0.054*
RE	85.0 ± 28.2	80.6 ± 30.0	2.454	0.013*
SF	85.7 ± 17.7	82.8 ± 18.7	2.597	0.005*
MH	88.7 ± 13.3	89.0 ± 13.4	−0.385	0.465
BP	86.4 ± 17.2	82.4 ± 18.4	3.626	0.008*
VT	87.3 ± 15.1	85.9 ± 15.9	1.490	0.288
Domain				
PC	85.8 ± 14.0	85.4 ± 13.2	0.215	0.554
MC	86.7 ± 15.4	84.7 ± 15.2	1.475	0.487

* indicate significance at *p* < 0.05

Key: Scales; physical functioning (*PF*), role limitations due to physical problems (*RP*), bodily pain (*BP*), general health (*GH*), vitality (*VT*), social functioning (*SF*), role limitations due to emotional problems (*RE*), and mental health (*MH*). Domains: physical health component (*PC*) and mental health components (*MC*)

**Table 6:** One way ANOVA comparison of the Yoruba version of the SF-36 scales and domains by age group

	Age group						
	18-24	25-34	35-44	45-54	≥55		
	(x̄ ± SD) (*n* = 475)	(x̄ ± SD) (*n* = 447)	(x̄ ± SD) (*n* = 79)	(x̄ ± SD) (*n* = 51)	(x̄ ± SD) (*n* = 35)	F-ratio	*p*-value
Scale							
GH	89.5 ± 12.3	90.7 ± 10.7	78.1 ± 27.5	62.3 ± 23.9	64.8 ± 33.6	15.795	0.001*
PF	86.9 ± 19.7	85.3 ± 16.9	90.2 ± 13.8	84.2 ± 16.7	74.8 ± 20.1	3.418	0.009*
RP	86.1 ± 23.4	85.8 ± 23.6	88.61 ± 20.33	72.1 ± 33.8	74.3 ± 27.4	6.242	0.001*
RE	82.5 ± 29.6	84.9 ± 27.9	92.4 ± 18.5	68.0 ± 38.3	74.3 ± 28.1	6.926	0.001*
SF	84.5 ± 17.4	85.8 ± 17.8	88.3 ± 16.0	77.7 ± 22.5	73.2 ± 23.5	6.706	0.001*
MH	86.7 ± 14.4	90.5 ± 12.5	92.4 ± 11.1	88.1 ± 11.4	89.3 ± 11.8	6.341	0.001*
BP	83.8 ± 18.5	85.4 ± 17.2	88.0 ± 15.7	87.2 ± 15.6	79.6 ± 19.3	2.115	0.077
VT	83.0 ± 17.4	90.1 ± 12.5	91.8 ± 13.0	85.6 ± 14.9	85.1 ± 15.6	15.489	0.001*
Domain							
PC	86.9 ± 12.6	86.1 ± 14.7	85.0 ± 12.7	72.5 ± 14.7	85.6 ± 9.6	3.121	0.016*
MC	84.2 ± 15.3	87.8 ± 14.4	91.1 ± 12.2	79.8 ± 20.8	80.5 ± 17.7	8.741	0.001*

Level of significance was set at *p* < 0.05

Key: Scales: physical functioning (*PF*), role limitations due to physical problems (*RP*), bodily pain (*BP*), general health (*GH*), vitality (*VT*), social functioning (*SF*), role limitations due to emotional problems (*RE*), and mental health (*MH*). Domains; physical health component (*PC*) and mental health components (*MC*)

The correlation between domains and scales of the Yoruba version of SF-36 (internal consistency of the scales and domains) are presented in Table 7. The result shows that correlations between scales and hypothesized domains (PC and MC) were above 0.50 (except the correlations between GH and each of PC (*r* = 0.477) and MC (0.28). The highest scale-domain correlation was between RE and MC (*r* = 0.826) and RP and MC (0.826). PC was strongly correlated (≥0.70) with each of PF (*r* = 0.711), RP (0.823) and BP (0.700) while MC was strongly correlated with each of RE (0.826), SF (0.811), MH (0.789) and VT (0.793). Intra-Class Correlation (ICC) of scores on the Yoruba SF-36 on two occasions (test-retest reliability) (*n* = 249) is presented in Table 8. The ICC ranges between 0.636 and 0.843 for scales, and between 0.783 and 0.851 for domains.

**Table 7:** Correlation of physical health components and mental health domains (in horizontal axis) with the 8 scales (vertical axis) in Yoruba version of SF-36 (*n* = 1087)

	Level of association		Value of correlation	
Scales	Physical health component	Mental health component	Physical health component	Mental health component
GH	*	-	0.477	0.280
PF	+	*	0.711	0.516
RP	+	*	0.823	0.545
RE	*	+	0.549	0.826
SF	*	+	0.660	0.811
MH	*	+	0.520	0.789
BP	+	*	0.700	0.612
VT	*	+	0.562	0.793

+: strong association (*r* > 0.70); *: moderate association (0.30 < *r* < 0.70); − :weak association (*r* < 0.30). Key: Scales: physical functioning (*PF*), role limitations due to physical problems (*RP*), bodily pain (*BP*), general health (*GH*), vitality (*VT*), social functioning (*SF*), role limitations due to emotional problems (*RE*), and mental health (*MH*). Domains: physical health component (*PC*) and mental health components (*MC*)

**Table 8:** Intra-Class Correlation of scores on the Yoruba SF-36 on two occasions (test-retest reliability) (*n* = 249)

	ICC	95 % confidence interval		*p*-value
		Lower bound	Upper bound	
Scale				
GH	0.636	0.451	0.768	0.001*
PF	0.802	0.747	0.846	0.001*
RP	0.785	0.732	0.829	0.001*
RE	0.715	0.647	0.771	0.001*
SF	0.777	0.722	0.823	0.001*
MH	0.843	0.802	0.876	0.001*
BP	0.768	0.710	0.815	0.001*
VT	0.796	0.745	0.838	0.001*
Domain				
PC	0.783	0.657	0.866	0.001*
MC	0.851	0.813	0.883	0.001*

Key: Scales; physical functioning (*PF*), role limitations due to physical problems (*RP*), bodily pain (*BP*), general health (*GH*), vitality (*VT*), social functioning (*SF*), role limitations due to emotional problems (*RE*), and mental health (*MH*). Domains; physical health component (*PC*) and mental health components (*MC*)

## Discussion

Translation and cross-cultural adaptation of HRQoL tools to languages other than the original population from which the tool was developed enhances understanding and facilitate acceptance of the tool by the accessible population [31–33]. This study was conducted to cross-culturally adapt the SF-36 into Yoruba language and determine its reliability and validity. A response rate of 98.8 % was achieved in this study, therefore, suggesting that the Yoruba SF-36 was an acceptable tool for measuring HRQoL in the Yoruba population. Based on difficulty and quality rating, the Yoruba SF-36 had a high rate of data completion with good quality data in the study population.

The concurrent validity of the Yoruba SF-36 was high, with scales and domains having co-efficient ranges greater than 0.70 that was considered desirable for good validity of a new tool. The correlation co-efficient ranges for concurrent validity obtained in this study, is consistent with scales and domains ranges of 83.2 to 88.8; and 85.6 and 85.9 respectively reported in previous studies [34–41]. From this study, all scale scores showed negative skewness among the sample population, implying that respondents gave answers that tilt towards the positive end of the health spectrum. Skewness distribution of the Yoruba SF-36 scales follows a similar pattern to previous findings on SF-36 in Hong Kong [36], Australia [37], Netherland [30], New Zealand [41], Brazil [42], Malaysia [20] and Turkey [16] among others.

The result of test of the known-group validity of the Yoruba SF-36 indicated that many dimensions of the SF-36 are influenced by socio-demographic variables such as age and gender. Men had higher mean scores in all scales (except MH) and domains. This finding is consistent with previous reports [11, 14, 39, 42]. However, the reason for higher HRQoL scores in men is still a subject of debate. Hopman et al. [39] implicated poorer HRQoL scores among women on higher incidences of psychological symptoms and greater psychological distress compared with men; in addition, women are more expressive of their symptoms and well-being. The finding of this study also showed that all the scales (except BP) and domains were associated with age. There was an obvious decline in mean score with older age across different scales and domains except within the 35–44 years age bracket. The result also revealed that that subscales of PC (i.e. PF, GH, BP and RP) decreased with older age, while age seem to have less influence on subscales of MC (MH, RE, SF and VT). This finding is also consistent with earlier reports [35, 37, 39, 41, 42].

The finding of this study showed a high level of item-scale correlations (i.e., correlations of an item with its own scale) greater than minimum value of 0.4 recommended by Ware et al. [10]. The finding showed that definite scaling success was met because the difference between the item-hypothesized scale and item-other scale correlation were >2 S.D. (i.e. >0.15) as recommended [10]. All items in the Yoruba SF-36 correlated strongly with its hypothesized scale than with scales measuring other concepts except the correlation of item 1 (i.e.” in general would you say your health is?”) of GH with RE, SF, BP and VT scales. Also, item-scale correlations were comparable within each scale, except item 1 which is similar to the findings of a previous study by Sararaks et al. [20]. Therefore, the pattern of item-scales correlation in this study was consistent with the recommendations for good psychometric criteria for SF-36 translations and cultural adaptions [31, 32, 34–41]. In addition, the test-retest results of the Cronbach’s α and ICC confirm high reliability of the Yoruba SF-36 at the level of scales and domains, greater than 0.7 coefficient level for good reliability for group-level analyses [8, 42–44].

This study’s findings on concurrent and discriminant validity, reliability and internal consistency indicates that the Yoruba SF-36 is a valid tool to assess HRQoL among Yoruba populace. The Yoruba SF-36 showed excellent psychometric properties comparable to the original American and other versions. However, item 1 (“In general would you say your health is?”) was poor on discriminant validity scores. Caution is recommended in the interpretation of the finding on item ‘1’ pending further studies. To validate the findings on the Yoruba SF-36 obtained in this study, further studies among various in health and disease populations are needed. The heterogeneity of sample population, mixed methods of sampling and using distance between two adjacent electric poles as equivalence of one block are potential limitations of this study.

## Conclusion

The data quality, concurrent and discriminant validity, reliability and internal consistency of the Yoruba version of the SF-36 are adequate and it is recommended for measuring health-related quality of life among Yoruba population.

## Appendix

### Yoruba Version of the SF-36 (ÌWÉ ASE ÌWÁDÌÍ ÌLERA - SF-36)

ÌLÀNÀ: Àtòjọ ìbéèrè wọ̀nyí fẹ́ mọ̀ nípa ìhà tí o kọ sí ìlera rẹ. Ìlànà ìbéèrè wọ̀nyí yóò fi bí ìlera rẹ ṣe rí hàn àti bí o ṣe ní ìlera láti máa ṣe àwọn ohun àmúṣe rẹ gbogbo. Dáhùn gbogbo ìbéèrè nípa títọ́ọka sí ìdáhùn gẹ́gẹ́ bó ṣe yẹ. Bí àtidáhùn ìbéèrè kan bá rú ọ lójú, pèsè ìdáhùn tó yẹ jù lọ bí o ṣe yé ọ sí.

1. (Jọ̀wọ́ ṣàmì sí àkámọ́ ìdáhùn **kan**) Lákòótán, ǹjẹ́ o lè sọ pé ìlera rẹ
2. Ní àfiwé pẹ̀lú ọdún kan sẹ́yìn, báwo ni o ṣe lè gbé ìlera rẹ lé orí òṣùwọ̀n lápapọ̀ báyìí? (Jọ̀wọ́ ṣàmì sí àkámọ́ ìdáhùn **kan**)
3. Àwọn ìbéèrè wọ̀nyí dá lórí ohun àmúṣe tí ó lè ṣe ní ọjọ́ kọ̀ọ̀kan. Ǹjẹ́ ìlera rẹ báyìí ń se ìdíwọ́ fún ọ láti se àwọn ohun àmúṣe wọ̀nyí? Bó bá rí bẹ́ẹ̀, báwo ló ṣe díwọ̀n tó? **(Jọ̀wọ́ yí òdo sí nọ́ḿbà kan lórí ìlà kọ̀ọ̀kan)**

4. Láàárín ọ̀sẹ̀ mẹ́rin sẹ́yìn, ǹjẹ́ o ní àwọn ìṣòro wọ̀nyí pẹ̀lú iṣẹ́ rẹ tàbí àwọn ohun àmúṣe ojoojúmọ́ mìíràn látàrí ìlera afojúrí rẹ? (**Jọ̀wọ́ yí òdo sí nọ́ḿbà kan lórí ìlà kọ̀ọ̀kan)**

5. Láàárín ọ̀sẹ̀ mẹ́rin sẹ́yìn, ǹjẹ́ o ní èyíkéyìí nínú ọ̀gọ̀ọ̀rọ̀ ìṣòro wọ̀nyí pẹ̀lú iṣẹ́ rẹ tàbí àwọn ohun àmúṣe ojoojúmọ́ mìíràn látàrí àwọn ìṣòro tó jẹ mọ́rònú (bi àpẹẹrẹ: níìrẹ̀wẹ̀sì tàbí hílàhílo)? (**Jọ̀wọ́ yí òdo sí nọ́ḿbà kan lórí ìlà kọ̀ọ̀kan**)

6. Láàárín ọ̀sẹ̀ mẹ́rin sẹ́yìn, báwo ni ìlera afojúrí rẹ tàbí àwọn ìṣòro tó jẹ mọ́rònú ṣe nípa tó lórí ìbásepọ̀ rẹ pẹ̀lú ẹbí, ọ̀rẹ́, alábàágbépọ̀ tàbí àwọn ẹgbẹ́ mìíràn? (Jọ̀wọ́ sàmì sí àkámọ́ ajẹmọ́dàáhùn **kan**)

7. Láàárín ọ̀sẹ̀ mẹ́rin sẹ́yìn, báwo ni ìrora ara tí o ní ṣe pọ̀ tó? (Jọ̀wọ́ ṣàmì sí àkámọ́ ajẹmọ́dàáhùn kan)

8. Láàárín ọ̀sẹ̀ mẹ́rin sẹ́yìn, báwo ni ìrora ṣe nípa lórí iṣẹ́ òòjọ́ rẹ sí (Tó fi mọ́ iṣẹ́ tòde àti ilé)? (Jọ̀wọ́ ṣàmì sí àkámọ́ ajẹmọ́dàáhùn **kan**)

9. Àwọn ìbéèrè wọ̀nyí dá lórí ìmọ̀lára rẹ àti bí àwọn nǹkan ṣe rí fún ọ láàrin ọ̀sẹ̀ mẹ́rin sẹ́yìn. (Jòwọ́ fúṇ wa ní ìdáhùn tó ṣúnmọ́ bí ìmọ̀lára rẹ ṣe rí jù lọ sí ìbéèrè kọ̀ọ̀kan)

10. Láàárín ọ̀sẹ̀ mẹ́rin sẹ́yìn, báwo ni àwọn ìṣòro afojúrí tàbí àwọn ìṣòro tó jẹ mọ́rònú rẹ ṣe nípa tó lórí ohun àmúṣe àwùjọ rẹ (gẹ́gẹ́ bí i bíbẹ àwọn ọ̀rẹ́ àti mọ̀lẹ́bí wò, àti bẹ́ẹ̀ bẹ́ẹ̀ lọ.) (Jọ̀wọ́ ṣàmì sí àkámọ́ ajẹmọ́dàáhùn **kan**)

11. Báwo ni ọ̀kọ̀ọ̀kan àwọn ìpèdè wọ̀nyí ṣe jẹ́ IRỌ́ tàbí ÒTÍTỌ́ fún ọ sí? (Jọ̀wọ́ yí òdo sí nọ́ḿbà kan lórí ìlà kọ̀ọ̀kan)

Ẹ ṣé!
